# Quantitative evaluation of corneal epithelial edema after cataract surgery using corneal densitometry: a prospective study

**DOI:** 10.1186/s12886-018-0998-5

**Published:** 2018-12-20

**Authors:** Sho Ishikawa, Naoko Kato, Masaru Takeuchi

**Affiliations:** 1 Department of Ophthalmology, Saitama Medical University, 38 Morohongo, Moroyama, Saitama, 350-0495 Japan; 2 Department of Ophthalmology, National Defense Medical College, 3-2 Namiki, Tokorozawa, Saitama, 359-8216 Japan

**Keywords:** Densitometry, Corneal edema, Cataract surgery

## Abstract

**Purpose:**

The optical density of the cornea can be evaluated quantitatively by “densitometry” using a rotating Scheimpflug camera. Densitometry allows evaluation of corneal opacity in the anterior segment of the eye by quantitative measurement of scattering light. In the present investigation, we evaluate quantitatively minimal subclinical corneal edema after cataract surgery using densitometry.

**Methods:**

Fifty four eyes of 34 patients who underwent cataract surgery were enrolled. Measurement of corneal density was performed using Pentacam® before and on days 1, 3 and 7 after surgery.

**Results:**

Densitometry scores increased from 18.12 ± 1.76 before cataract surgery to 21.03 ± 3.84 on day 1 (*P* < 0.001) and 19.90 ± 2.46 on day 3 (*P* = 0.018), but recovered to 19.44 ± 1.58 on day 7 (*P* = 0.131). Total corneal thickness was 549.1 ± 32.7 μm before surgery and increased to 582.7 ± 46.3 μm on day 1 (*P* = 0.001), but recovered to 566.4 ± 29.7 μm on day 3 (*P* = 0.097). Densitometry reading correlated positively with corneal thickness (correlation coefficient = 0.13, *P* = 0.003).

**Conclusions:**

Densitometry is useful to detect corneal edema that is not detectable by slit-lamp examination.

**Electronic supplementary material:**

The online version of this article (10.1186/s12886-018-0998-5) contains supplementary material, which is available to authorized users.

## Introduction

The Pentacam® (Oculus, Wetzlar, Germany), a corneal tomography using a rotating Scheimpflug camera is a diagnostic tool for the anterior segment of the eye, allows quantitative evaluation of the optical media as “densitometry”. The densitometry program that measures scattering light allows quantitative and objective measurement of opacities within the anterior segment of the eyes. Since Smith et al. [1] first reported on the evaluation of healthy cornea by Scheimpflug imaging, densitometry has been used for objective assessment of cataract-associated media opacification.

Several clinical studies have reported the usefulness of densitometry for quantitative evaluation of corneal opacification in various conditions such as corneal dystrophy [2], mucopolysaccharidosis [3], corneal haze after corneal cross linking for keratoconus [4, 5] or keratectasia [4], laser in situ keratomileusis [6, 7], photorefractive keratectomy [8, 9], and lamellar keratoplasty [10, 11], corneal infection [12], after Descemet’s stripping automated endothelial keratoplasty [13] or penetrating keratoplasty [14], increase in corneal light scattering has been detected by densitometry even in cases clinically assessed to be clear by slit-lamp microscopy, indicating the potential use of densitometry for objective measurements as an adjuvant to slit-lamp examination. However, there is no report that densitometry can be used as an indicator of minimal corneal edema.

Phacoemulsification is a standard procedure for cataract surgery, and is the most common procedure performed in recent decades. Even in uneventful cataract surgeries, various intraoperative factors such as ultrasound energy emitted by phacoemulsification, greater infusion volume, mechanical contact of nuclear fragments with the corneal endothelium, and high intraocular pressure may cause subclinical corneal edema, although no significant findings are detected by slit-lamp examination.

The purpose of the present study was to detect quantitatively serial changes of backward light scattering after cataract surgery using the densitometry.

## Materials and methods

Fifty four eyes of 34 patients (aged 71 ± 8.4 years, 18 males and 16 females) who underwent cataract surgery at the Department of Ophthalmology, National Defense Medical College between July 2012 and October 2013 were enrolled; a prospective study. All eyes had nuclear cataract of grade 2 or above were included. Eyes with ocular surface diseases before surgery, such as dry eye and superficial punctate keratopathy, were excluded.

In addition to routine ophthalmic examinations, we assessed corneal densitometry readings and corneal thickness by Pentacam® before cataract surgery and on days 1, 3 and 7 after surgery. Examination with Pentacam® was conducted as recommended in the instruction manual. After the patient had placed his/her head on the instrument’s head and chin rests, the Pentacam® was aligned so that a dim, blue light illuminated the cornea. The instrument was aligned so that the patient’s eye and the live Scheimpflug image came into view. The apex of the cornea was marked by positioning a yellow circle, and the patient was asked to open the eye widely. Twenty-five Scheimpflug images were taken automatically in three-dimensional scan mode. The instrument allows full-thickness corneal haze evaluation, and shows the measured maximum density values on a densitogram on a scale from 0 to 100; 0 indicating no clouding and 100 indicating completely opaque.

**Fig. 1:**
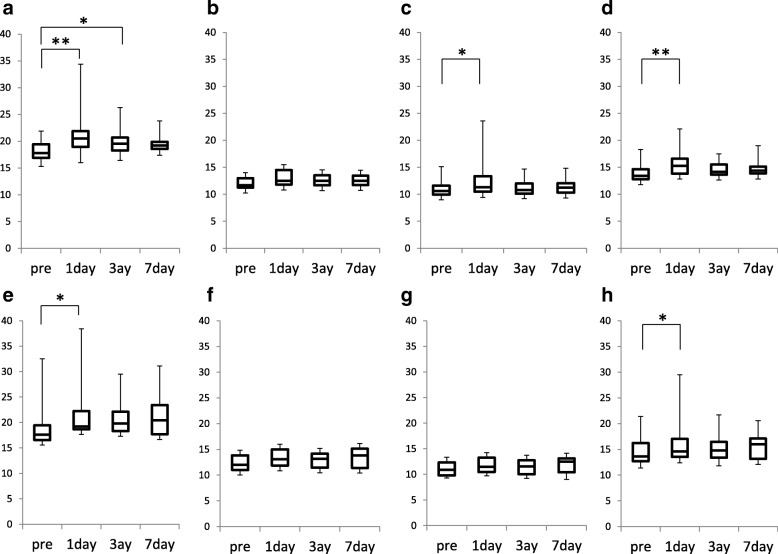
Changes in densitometry readings before and 1, 3 and 7 days after cataract surgery. **a** to **d**: Corneal densitometry readings in the central 2 mm zone for the anterior layer (120 μm) (**a**), center layer (**b**), posterior layer (60 μm) (**c**), and total depth (**d**). **e** to **h**: Corneal densitometry readings in the 2–6 mm annulus zone for the anterior layer (**e**), center layer (**f**), posterior layer (**g**), and total depth (**h**). *p < 0.05, **p < 0.01 versus preoperative reading by Friedman’s test

We measured corneal density with the cornea density mode of Pentacam®. The cornea is divided into 12 portions according to the zone and the depth; namely, four zones in concentric circles from the corneal apex (central 2 mm zone, surrounding 2–6 mm annulus, 6–10 mm annulus, and 10–12 mm annulus) and three depths (anterior layer: from the corneal surface to a depth of 120 μm, center layer: between the anterior and posterior layers, and posterior layer: the deepest 60 μm). The densitometry value is expressed as the average score in each portion. We used the corneal densitometry score in the central 2 mm zone and the anterior layer (0–120 μm depth) for analysis (Fig. 1). Before the surgery, the lid skin was scrubbed with 10% povidone-iodine (Meiji Seika, Tokyo, Japan) and the conjunctiva and cornea within the operative field were disinfected with 1% povidone-iodine. For topical anesthesia, 4% lidocaine eye drops were instilled onto the operated eye. All cataract surgeries were performed by the same surgeon (S.I) using phacoemulsification and aspiration technique via a small incision (2.4 mm). The Infiniti® (Alcon, Fort Worth, Texas) was used as the phacoemulsification instrument. The setting of the instrument was as follows: irrigation pressure 80–90 cmH2O, aspiration rate 20–25 mm/min, aspiration pressure 40–350 mmHg, and phacopower 40–60%.

The study protocol was approved by the ethical committee of the National Defense Medical College and the research was conducted according to the tenets of the Declaration of Helsinki.

### Statistical analysis

JMP software version 10 was used for the statistical analysis. Friedman’s test with Bonferroni correction as multiple comparison procedure was used as a nonparametric test to compare corneal densitometry score and corneal thickness. The Spearman’s correlation coefficient was used to analyze correlation between corneal densitometry score and corneal thickness. *P* values less than 0.05 were considered statistically significant.

## Results

Fifty four eyes of 34 patients (aged 71 ± 8.4 years, 18 males and 16 females) were enrolled. All eyes had nuclear cataract of grade 2 or above. Sixteen eyes had posterior subcapsular opacity, 34 eyes had cortical opacity, and 4 eyes had mature cataract. All cataract surgeries were completed uneventfully, and no perioperative complications were observed. Best corrected visual acuity (BCVA) was improved in all eyes. The average BCVA (Log MAR) was 0.33 ± 0.38 before surgery and 0.05 ± 0.19 on day 1 after surgery. Intraocular pressure was within normal range in all eyes before (10–19 mmHg) and after (7–17 mmHg) surgery (Table 1). Slit-lamp examination detected minimum fold of the Descemet’s membrane after surgery in 6 of 54 eyes.

**Table 1:** Changes in BVCA, corneal thickness, and intraocular pressure

	Preoperative	Postoperative		
		Day1	Day3	Day7
BVCA (LogMAR)	0.332 ± 0.382	0.050 ± 0.186*		0.008 ± 0.151*
Central corneal thickness (μm)	550.1 ± 32.4	588.3 ± 53.9**	567.8 ± 29.9	561.1 ± 31.9
Intraocular pressure(mmHg)	14.2 ± 2.6	13.2 ± 3.1	11.5 ± 3.1*	12.0 ± 2.5*

*BCVA* best corrected visual acuity

*p < 0.05, **p < 0.01 versus preoperative, by Friedman’s test

**Fig. 2:**
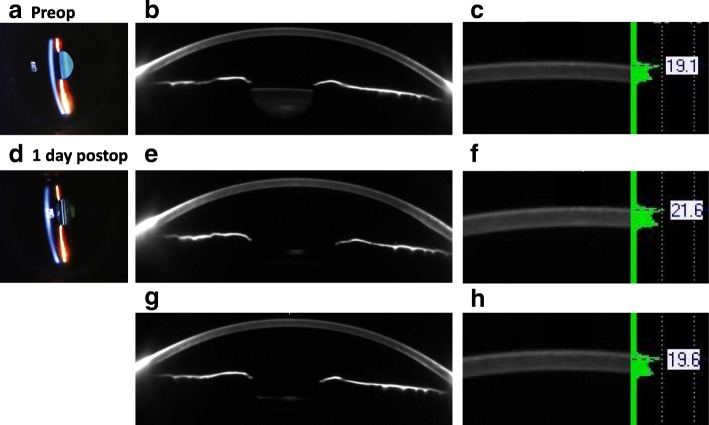
Representative slit-lamp photographs (left), Scheimpflug images (center), and densitometry readings (right) of central cornea before cataract surgery (**a**) and on day 1 (**b**) and day 3 after surgery (**c**). Slit-lap examination reveals no abnormal finding before (**a**) and 1 day after surgery (**b**). Scheimpflug images show minimal opacity on day 1 and day 3 after surgery. Densitometry score is 19.1 before cataract surgery (**a**), increases to 21.6 on day 1 (**b**) and recovers to 19.6 on day 3 after surgery (**c**)

Densitometry readings in the central 2 mm zone within the anterior layer was 18.12 ± 1.76 before surgery, and increased significantly to 21.03 ± 3.84 on day 1 (*P* < 0.001) and 19.90 ± 2.46 on day 3 (*P* = 0.017) but recovered to 19.44 ± 1.58 on day 7 (*P* = 0.131) after surgery (Fig. 1a). Densitometry readings in the 2–6 mm annulus within the anterior layer was 19.11 ± 4.66 before surgery and increased significantly to 22.18 ± 7.40 on day 1 (*P* = 0.046) but declined to 20.73 ± 4.60 on day 3 (*P* = 0.296) and 21.39 ± 4.86 on day 7 (*P* = 0.183) after surgery (Fig. 1e). In the center layer, the densitometry readings showed no significant changes both in the central 2 mm zone and the 2–6 mm annulus throughout the study period (Fig. 1b and f). In the posterior layer, densitometry readings in the central 2 mm zone increased significantly only on day 1 after surgery (10.8 ± 1.3 to 12.4 ± 2.9, *P* = 0.003), but returned to preoperative value on day 3 after surgery (Fig. 1c and g, Fig. 2, Additional file 1).

**Fig. 3:**
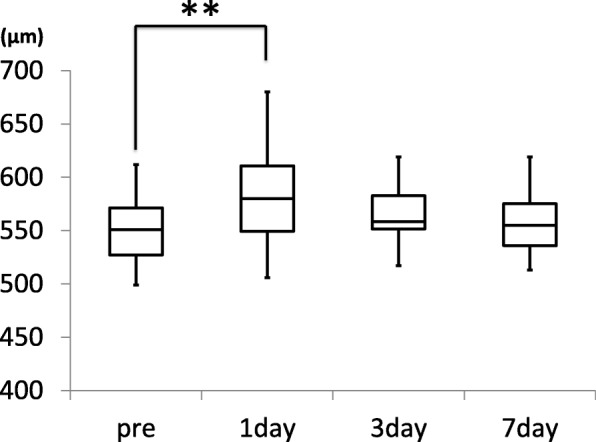
Changes in central corneal thickness. Total corneal thickness at the corneal apex is 549.1 ± 32.7 μm before surgery and increases to 582.7 ± 46.3 μm on day 1 after surgery (*P* = 0.001 vs. preoperative). Corneal thickness recovers to 566.4 ± 29.7 μm on day 3 (*P* = 0.097 vs. preoperative) and 559.4 ± 32.4 μm on day 7 (*P* = 0.400 vs. preoperative) after surgery

Total corneal thickness (mean ± standard deviation) at the corneal apex was 549.1 ± 32.7 μm before surgery and increased to 582.7 ± 46.3 μm on day 1 after surgery (*P* = 0.001), but recovered to 566.4 ± 29.7 μm on day 3 (*P* = 0.097) and 559.4 ± 32.4 μm on day 7 (*P* = 0.400) after surgery (Fig. 3).

**Fig. 4:**
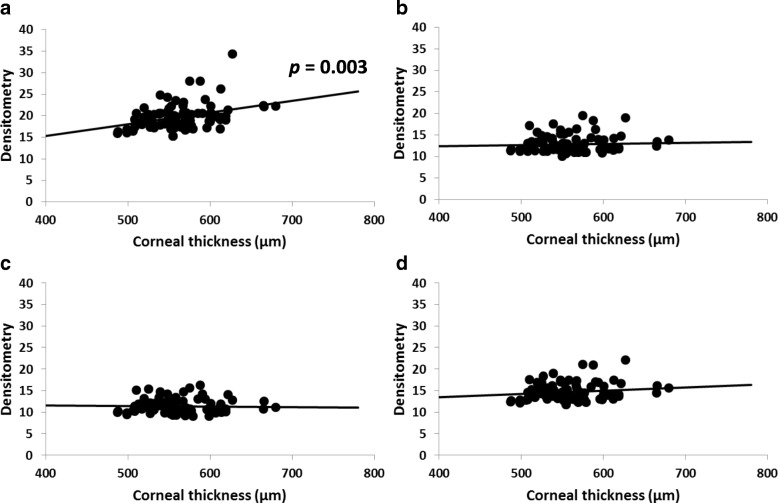
Correlation of corneal thickness with densitometry score for the anterior layer (**a**), center layer (**b**), posterior layer (**c**), and total depth (**d**)**.** Densitometry reading for the anterior layer correlates positively with central corneal thickness [correlation coefficient (r) = 0.36, *P* = 0.003]. No correlation is observed for the center layer (**b**; *r* = 0.05, *p* = 0.87), the posterior layer (**c**; *r* = 0.02, *p* = 0.47), and total depth (**d**; *r* = 0.14, *P* = 0.44), * *P* < 0.05 by Spearman’s correlation coefficient

Densitometry readings in the central 2 mm zone for the anterior layer correlated positively with the corneal thickness (correlation coefficient = 0.36, *P* = 0.003), but the densitometry readings for the center and posterior layers and total depth did not correlate significantly with the corneal thickness (Fig. 4).

## Discussion

In the present investigation, we used a rotating Scheimpflug camera to assess the serial changes of corneal densitometry from before cataract surgery to 7 days after surgery. Our results showed transient increases in densitometry reading after uneventful cataract surgeries and a significant correlation between densitometry readings and corneal thickness. These findings may indicate that subclinical corneal epithelial or stromal edema occurs and causes light scattering shortly after cataract surgery.

In this study, densitometry readings in the anterior layer were higher than that in the posterior layer. A similar tendency was also reported in previous investigations using confocal microscopy [15] or scatterometer [16]. According to previous evaluations using atomic force microscopy [17] and second harmonic generation microscopy [18, 19], collagen fibers are interwoven in three dimensions and adhere densely to Bowman’s layer and fiber density is less dense in the anterior stroma than in the posterior stroma in healthy cornea [18]. The difference in densitometry readings between anterior and posterior corneal layer may reflect the difference in stromal collagen structure between different layers.

Ní Dhubhghaill et al. [20] evaluated corneal densitometry in normal Caucasian eyes using Scheimpflug camera. According to their study, densitometry reading in the central 2 mm zone in the anterior layer (up to 120 μm in depth) was 22.87 ± 2.91, and densitometry reading in the central 2 mm zone for total depth was 16.76 ± 1.87. In the present study, however, the densitometry reading in the central 2 mm zone was 18.1 ± 1.8 in the anterior layer, and was 14.0 ± 1.7 for total corneal depth. We speculate that the difference in scores may reflect racial difference, because all patients enrolled in our study were Asian. Corneal thickness was reported to differ between races, and the central corneal thickness is thicker in Caucasian eyes compared to Asian eyes [21–24]. Age also changes the value of densitometry. The densitometry readings of central and posterior layer increased with age but not anterior layer [25]. According to their study, densitometry reading of 60–69 years old in the central 2 mm zone in the anterior layer was 18.31, center was 15.21, posterior was 12.23 and total depth was 15.37 in Caucasian eyes. In this study, light scattering may increase when the number of corneal collagen fibers is larger, possibly resulting in increase in densitometry reading. It is reported that the ability to detect changes in backscatter was superior with Scheimpflug camera to confocal microscopy [26].

Another possibility is that we excluded eyes with corneal surface diseases including dry eye and superficial punctate keratopathy both before and after the surgery in the present investigation. Such ocular surface distintegrity could cause light scattering and possibly influence the densitometry readings. The densitometry readings might increase due to corneal epithelial damage, while increase of densitometry readings in the anterior layer cannot distinguish the light scattering of the epithelial or superficial stromal layer. There are some reports that some drugs using during surgery caused corneal epithelial damage. 5% povidone iodine may cause corneal epithelial damage [27] and 10% povidone iodine with presurgical skin antiseptics are toxic for cornea [28]. We used 1% povidone iodine the conjunctiva and cornea within the operative field, but 10% povidone iodine was used with skin. We cannot deny possibility that 10% povidone iodine has exposed to the cornea and affected densitometry readings. Topical anesthesia may also corneal epithelial damage. When the cornea becomes edematous, intra- and inter-cellular fluid accumulation occurs in the epithelial layer, and is followed by subsequent accumulation of fluid within and between the stromal lamellae within the underneath stroma. These edematous changes of epithelial and underneath stromal structure may cause light scattering, which is partially reflected by the increase in densitometry reading.

The correlation between corneal thickness and light scattering of the cornea was first reported in 1982 by Olsen [29], who applied slit-lamp fluorophotometry to measure light scattering of the whole cornea in eyes that underwent cataract surgery within 1 week prior to study. He speculated that light scattering increases sharply as the tissue swells; the space between collagen fibrils becomes less regular, thereby increasing the amount of light scattered. These findings may support our theory that the elevation of densitometry reading is due to increase in light scattering caused by corneal epithelial and stromal edema, although the surgical procedure used in Olsen’s study was supposed to be extracapsular extraction, which is more invasive than the small incision phacoemulsification used in the present study.

Interestingly, corneal edema occurred shortly after surgery not only around the incision but also in central cornea. However, the edema was transient and improved quickly in subsequent several days. The densitometry readings in the central 2 mm zone improved faster than in the surrounding 2–6 mm zone, probably reflecting centripetal processes of wound healing [30, 31] and endothelial cell migration [32]. Further investigations on the topographical changes of densitometry reading and corneal edema may contribute to elucidate the mechanisms of water distribution and its relation with wound healing in the cornea.

This study had some limitations. First, we did not use confocal microscopy in this study, so we cannot confirm more detailed corneal change. Second, we did not assessed quality of vision such as glare, halos and blurring. None of the patients complained symptoms after the surgery, but there is a possibility that patient did not feel symptoms the several days after surgery. Third, corneal densitometry readings of posterior segment are influenced from the anterior corneal haze. As the anterior densitometry readings increases, the values of central and posterior densitometry readings may be inaccurate. In this study, there was no patient who had very high densitometry readings compared to previous reports such as corneal surgery [8–11], densitometry readings of anterior segment may influence the readings of central and posterior segment.

## Conclusions

In summary, densitometry using the Scheimpflug device for measuring corneal scattering may be a useful tool to detect minimal subclinical corneal edema usually undetectable by slit-lamp examination. There is a possibility that densitometry helps to detect subtle edema of the cornea particularly in experimental studies.

## Additional file

Additional file 1: Corneal densitometry readings pre and postoperative surgery. Densitometry readings in the central 2 mm zone within the anterior layer before and postoperative on day 1, day 3 and day 7 were 18.1 ± 1.8, 21.3 ± 3.8, 19.9 ± 2.5, 19.4 ± 1.6. Densitometry readings in the 2–6 mm annulus within the anterior layer before and postoperative on day 1, day 3 and day 7 were 19.1 ± 4.7, 22.1 ± 7.4, 20.7 ± 4.6, 21.3 ± 4.9. Densitometry readings in the central 2 mm zone within the center layer before and postoperative on day 1, day 3 and day 7 were 12.2 ± 1.5, 13.7 ± 2.6, 12.8 ± 1.5, 13.0 ± 1.8. Densitometry readings in the 2–6 mm annulus within the center layer before and postoperative on day 1, day 3 and day 7 were 12.6 ± 2.0, 14.2 ± 3.8, 13.1 ± 1.8, 13.5 ± 2.1. Densitometry readings in the central 2 mm zone within the posterior layer before and postoperative on day 1, day 3 and day 7 were 10.8 ± 1.3, 12.4 ± 2.9, 11.2 ± 1.4, 11.4 ± 1.5. Densitometry readings in the 2–6 mm annulus within the posterior layer before and postoperative on day 1, day 3 and day 7 were 11.3 ± 1.6, 12.3 ± 2.4, 11.5 ± 1.5, 11.9 ± 1.7. Densitometry readings in the central 2 mm zone before and postoperative on day 1, day 3 and day 7 were 14.0 ± 1.7, 15.7 ± 2.8, 14.6 ± 1.6, 14.8 ± 1.6. Densitometry readings in the 2–6 mm annulus before and postoperative on day 1, day 3 and day 7 were 14.6 ± 2.8, 16.4 ± 4.3, 15.1 ± 2.3, 15.6 ± 2.6. (DOCX 82 kb)

## Abbreviation

BCVA: Best corrected visual acuity
