# Supplementary material for: Quantitative evaluation of corneal epithelial edema after cataract surgery using corneal densitometry: a prospective study
Source: BMC Ophthalmol. 2018 Dec 20;18:334. doi: 10.1186/s12886-018-0998-5 (PMC6302480; doi:10.1186/s12886-018-0998-5)
Supplement: Supplementary file 1 — Corneal densitometry readings pre and postoperative surgery. Densitometry readings in the central 2 mm zone within the anterior layer before and postoperative on day 1, day 3 and day 7 were 18.1 ± 1.8, 21.3 ± 3.8, 19.9 ± 2.5, 19.4 ± 1.6. Densitometry readings in the 2–6 mm annulus within the anterior layer before and postoperative on day 1, day 3 and day 7 were 19.1 ± 4.7, 22.1 ± 7.4, 20.7 ± 4.6, 21.3 ± 4.9. Densitometry readings in the central 2 mm zone within the center layer before and postoperative on day 1, day 3 and day 7 were 12.2 ± 1.5, 13.7 ± 2.6, 12.8 ± 1.5, 13.0 ± 1.8. Densitometry readings in the 2–6 mm annulus within the center layer before and postoperative on day 1, day 3 and day 7 were 12.6 ± 2.0, 14.2 ± 3.8, 13.1 ± 1.8, 13.5 ± 2.1. Densitometry readings in the central 2 mm zone within the posterior layer before and postoperative on day 1, day 3 and day 7 were 10.8 ± 1.3, 12.4 ± 2.9, 11.2 ± 1.4, 11.4 ± 1.5. Densitometry readings in the 2–6 mm annulus within the posterior layer before and postoperative on day 1, day 3 and day 7 were 11.3 ± 1.6, 12.3 ± 2.4, 11.5 ± 1.5, 11.9 ± 1.7. Densitometry readings in the central 2 mm zone before and postoperative on day 1, day 3 and day 7 were 14.0 ± 1.7, 15.7 ± 2.8, 14.6 ± 1.6, 14.8 ± 1.6. Densitometry readings in the 2–6 mm annulus before and postoperative on day 1, day 3 and day 7 were 14.6 ± 2.8, 16.4 ± 4.3, 15.1 ± 2.3, 15.6 ± 2.6. (DOCX 82 kb) [file 12886_2018_998_MOESM1_ESM.docx]

Corneal densitometry readings pre and postoperative surgery
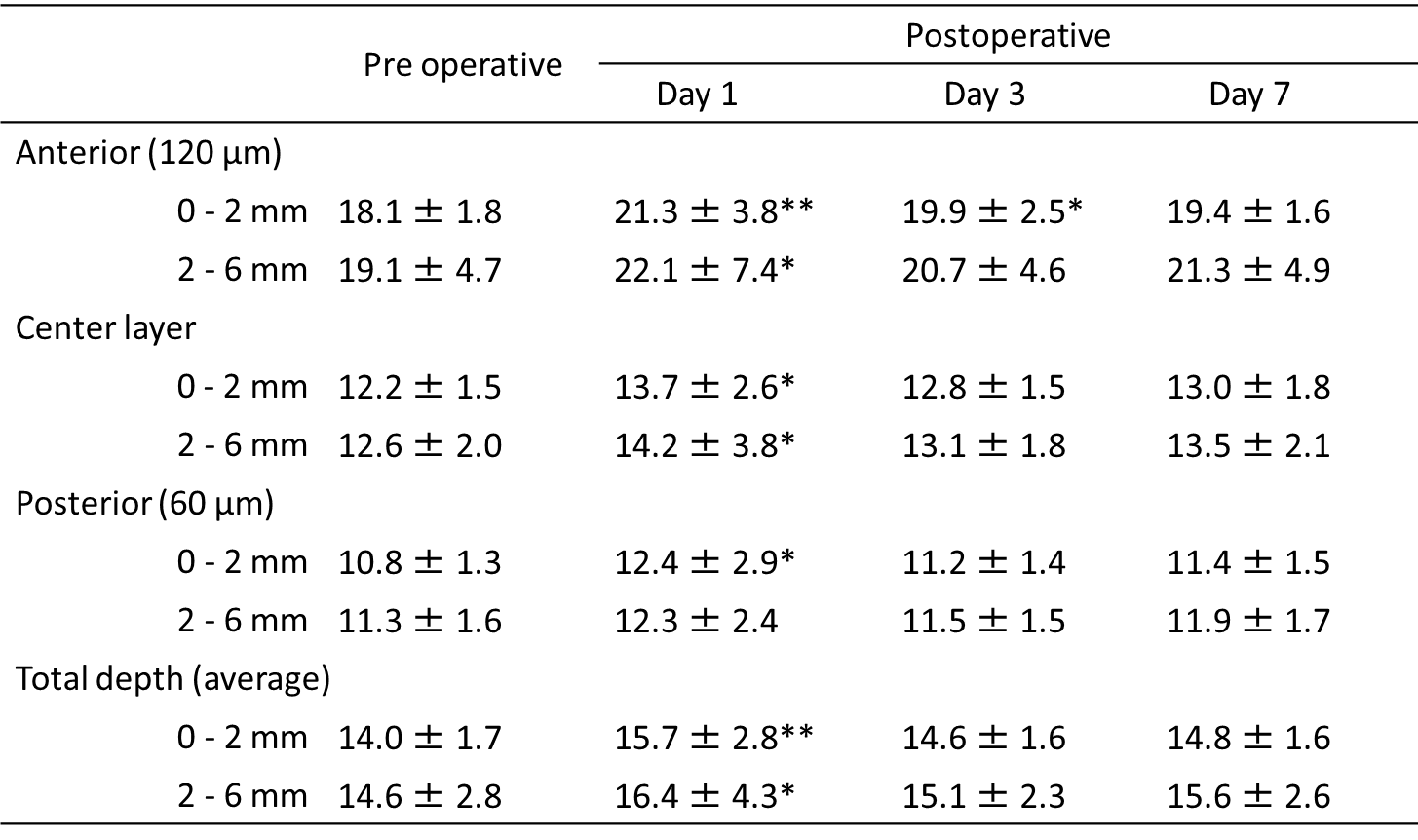


*p < 0.05, **p < 0.01 versus preoperative, by Friedman’s　test
